# Investigating incidence of bacterial and fungal contamination in shared cosmetic kits available in the women beauty salons

**DOI:** 10.15171/hpp.2016.25

**Published:** 2016-08-10

**Authors:** Leila Dadashi, Reza Dehghanzadeh

**Affiliations:** Department of Environmental Health Engineering, Faculty of Health, Tabriz University of Medical Sciences, Tabriz, Iran

**Keywords:** Shared cosmetic, Beauty salons, Microbial contamination, Women, Bacteria, Fungi

## Abstract

**Background:** Rich texture of cosmetics can provide a suitable medium for growth of pathogenic microorganisms. In addition, skin microflora of anyone is unique which might be harmful to another person. Skin and eye pathogenicity could be communicated by sharing cosmetics in beauty saloons. The main objective of this study was to evaluate microbial contamination of in-use skin and eye cosmetics which are available as public make-up kits for women in the beauty salons.

**Methods**: Fifty-two in-use skin and eye cosmetics were included in this cross sectional study.The specimens from all the cosmetics were collected following the owner’s informed consent, and then about 1 g of the cosmetics was added to nine ml of liquid Eugon LT100 broth medium,two for each product. Ten beauty salons randomly selected from different regions of Tabriz city between June and August 2016. Cosmetics were sampled and carried to the laboratory in sterile condition and then examined to determine bacterial and fungal species in the samples.

**Results:** All of in-use cosmetic were contaminated with bacteria (95% CI = 93.1%-100.0%) and about 19.2% by fungus and yeast (95% CI = 10.8%-31.9%). Streptococcus spp., Pseudomonas spp., Acinetobacter, Bacillus spp., Staphylococcus spp., Escherichia coli, Salmonella, Klebsiella,Citrobacter, Rhodotorula and Candida were dominant species which were isolated from the cosmetics. Powders with 38.5% (95% CI = 17.7%-64.5%) and eyeliners with 30.0% (95%CI = 6.7%-65.2%) were the most fungal contaminated products.

**Conclusion:** Shared cosmetics in beauty salons are almost contaminated by bacteria and fungus.Therefore, it is suggested to avoid sharing cosmetics by women and prevent use of public cosmetics in toilet saloons.

## Introduction

In recent years, cosmetics are extensively used for beauty purposes. Meanwhile, beauty salons play an important role in possible transfer of skin and eye infections due to the use of public make-up kits by the women.^1^ Although the microbial standards of cosmetics have been progressively improved by stringent legislations, their contamination has been frequently reported and even in some cases, has generated serious problems for consumers.^2^ Often production and expiration date are not labeled on the cosmetics, also effectiveness of cosmetic’s preservative decreases with time. In addition, cosmetics comprise essential minerals, growth factors, organic and inorganic compounds and humidity which provide suitable conditions for augmentation of microorganisms.^3^ Skin microflora of anyone is unique and could be transferred to the others by using common tools such as brushes and pads which could threaten the healthiness of the women.^4^ Therefore, it is likely that common cosmetics in beauty salons have more diversity and density of microorganisms.

Survey on personal toiletries show that *Bacillus, Staphylococcus* spp.,* Pseudomonas* spp., *Enterobacter*, *Aspergillus, Penicillium* and *Candida* are more predominant species in cosmetics.^3,5^ Also the most common skin infections are caused by *Staphylococcus epidermis* and * Staphylococcus aureus*.^6^ However, microbial contamination of in-use cosmetics in beauty salons could be hardly found in the literature and often brushes and combs and other similar devices have been surveyed. The most dominant isolated species from in-use tools in beauty salons have been *Streptococcus* spp*.*, *Staphylococcus* spp*.*, *Escherichia coli, Citrobacter freundi, Klebsiella, Enterobacter* and *Pseudomonas aeruginosa* and also fungus like *Aspergillus* and* Penicillium*.^1^ Cosmetic products can be contaminated by three ways; (1) application of unsterile raw material as ingredients, (2) in the course of production process, or (3) during use of cosmetics.^7^ On the other hand, trafficking counterfeit cosmetic products is the serious problem in many countries.

Consumption of cosmetics is growing in developing countries. Microbial contamination and occurrence of skin contamination due to cosmetics is still one of the major causes for product recalls in the world.^8^ Then, the main purpose of this study was to evaluate bacterial and fungal contamination of in-use eye and skin cosmetics shared by women in beauty salons.

## Materials and Methods

### Sampling

To determine the microbial contamination of in-use shared cosmetics available in the beauty salons, about 52 in-use skin (powder and cream) and eye (mascara and eyeliner) cosmetics were included in this cross-sectional study based on sample size calculation for dichotomous variable. The sample size was estimated about 61 (significance level=0.05, population proportion=0.2 and relative error=10%), but nine of the samples were discarded because of probably contamination during handling. The specimens from all the cosmetics were collected following the owner’s informed consent, and then about one gram of the cosmetics was added to nine ml of liquid *Eugon LT100* broth medium, two for each product. Ten beauty salons randomly selected from different reign of Tabriz city between June and August 2016. Sampling of cosmetics was conducted in the salons.

### Microbial survey

In sterile conditions, about 1 g of the cosmetics was added to nine ml of liquid *Eugon LT100* broth medium to neutralize the growth inhibitors present in the ingredients of the cosmetics. The samples immediately were carried to the laboratory and analyzed in accordance with the standards of Food and Drug Administration (FDA) and Institute of Standards and Industrial Research of Iran.^9^ First the tubes were incubated for 48-72 hours at 37°C. Then, 1 mL of each culture was removed and transferred to the Cetrimide Agar medium, Levine eosin methylene blue Agar medium, Baird Parker Agar, and Sabouraud Dextrose Chloramphenicol Agar and incubated for 24-48 hours at 37°C. Afterwards, the plates containing growing colonies were isolated and the total count of colony forming unit per gram or milliliter of cosmetics (CFU g^-1^) was determined by counting the colonies on the medias. Further identification of the isolated bacteria were carried out according to the bacteria’s morphology and biochemical tests using standard bacteriological methods.^10^ Fungi and molds were identified in terms of appearance. In addition, the relevant test to detecting *Candida* yeast including culturing in human serum and incubation at 37°C was conducted for 3 hours.^11^

### Statistical analyses

Variance between the contamination levels in the in-use cosmetics as well as between different cosmetic types was determined by chi-square k-sample Pearson analysis with significance level of 0.05 using SPSS software (IBM SPSS Statistics 19, SPSS Inc., USA). Confidence intervals (CI) were calculated by Stata MP 14 (Stata Corp LP, USA).

## Results

Table 1 shows that, exactly 100% (95% CI = 93.1%-100%) of the total examined in-use cosmetics in the beauty salons were contaminated by bacteria. However, only 19.2% (95% CI=10.8%-31.9%) of the cosmetic products were contaminated by fungi or yeast. Generally powders demonstrated higher contamination by fungi. The results show that creams did not indicated any contamination by fungi.

The number of colony forming units of fungi in cosmetics was between 3.5-200×10^3^ CFU g^-1^ (Table 2). Also the number of colony forming units of isolated bacteria was 12-960×10^3^ CFU g^-1^. High levels of *Staphylococcus* spp. and* Escherichia coli* counts (>500 CFU g^-1^) were found in the in-use powders and eyeliners.

**Figure 1:**
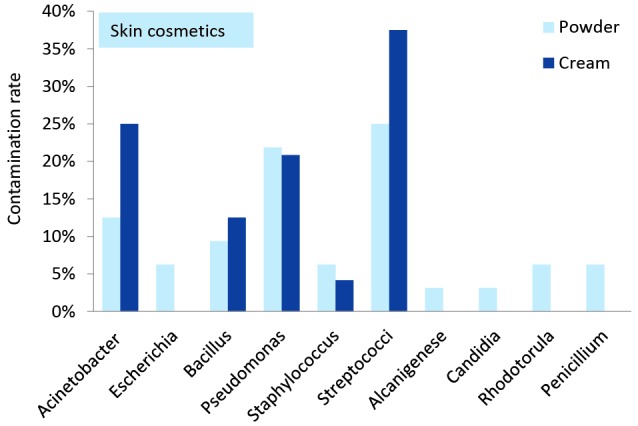
Microbial contamination rate in the in-use skin cosmetics in women beauty salons.

**Figure 2:**
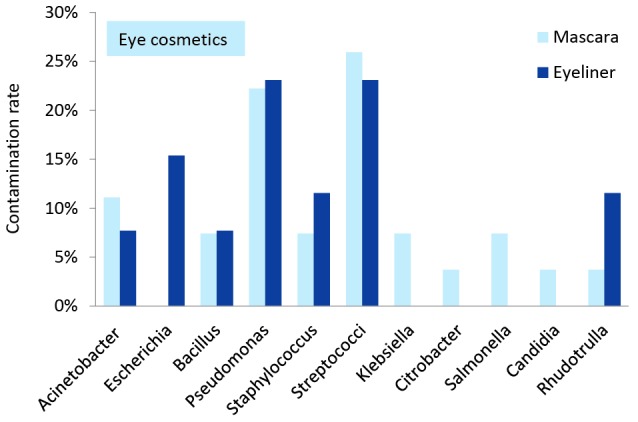
Microbial contamination rate in the in-use eye cosmetics in women beauty salons.

Figure 1 and 2 demonstrate the diversity and frequency of the isolated bacteria and fungi separately in skin and eye cosmetics obtained from beauty salons. Fungi and bacteria constituted 9.2% (95% CI=5.1%-16.1%) and 90.8% (95% CI=83.9%-94.9%) of the isolates, respectively. Also about 51.5% (95% CI=41.8%-61.1%) of the isolated bacteria were belong to gram-negative group and the remains were gram-positive. *Streptococcus* spp.*, Acinetobacter* and *Pseudomonas* spp. were the most dominant in the skin cosmetics. *Candida, Rhodotorula* and *Penicillium* were the only isolated yeasts and fungi. Also *Bacillus* spp.*, Staphylococcus* spp. and *Escherichia coli* isolated from the skin cosmetics. *Streptococcus* spp.*, Pseudomonas* spp. and *Acinetobacter* were the most frequently isolated bacteria from in-use eye cosmetics. *Rhodotorula* and *candida* were the only isolated yeasts. Also *Bacillus* spp, *Staphylococcus* spp.*, Escherichia coli, Salmonella, Klebsiella* and* Citrobacter* were isolated from the eye cosmetics.

*Streptococcus* species was the most predominant bacteria that were isolated from the in-use skin and eye cosmetics. Considering the fungi isolated from in-use skin powders, *Penicillium* was definitely the most predominant fungi genus (6%), which was followed in order by *Rhodotorula* (6%) and *Candida* (3%). Furthermore, *Rhodotorula* (12%) and *Candida* (4%) were the most isolated fungi from the in-use mascaras and eyeliners. Moreover, the most fungal diversity was observed in the in-use skin powders. Referring to the isolated bacteria from the in-use skin cosmetics, the most predominant bacteria were *Streptococcus* (32%), *Pseudomonas* (23%), *Acinetobacter* (19%)*, Bacillus* (11%), *Staphylococcus* (6%) and* E. coli* (4%). Among the in-use eye cosmetics, *Streptococcus* (25%) and *Pseudomonas* (24%) were the predominant isolated bacteria, which was followed in a descending order by *Acinetobacter* and* Staphylococcus* (10% each), *Bacillus* and* E. coli* (8% each), *Salmonella* and *Klebsiella* (4% each), and *Citrobacter* (2%).

**Table 1:** Summery of microbial contamination rate in the sampled cosmetics from women beauty salons

**Cosmetic type**	**No. of samples**	**Microbial contamination rate**					
		**Bacteria**			**Fungi**		
		**n (%)**	**95% CI**	***P value***	**n (%)**	**95% CI**	***P value***
Skin							
Powder	13	13(100.0 )	93.12-100.0	NS^a^	5(38.5)	17.7-64.5	
Cream powder	12	12(100.0)	75.8-100.0	NS	0.0	0.0-24.3	
Eye							0.063
Mascara	17	17(100.0)	81.6-100.0	NS	2(11.8)	3.3-34.3	
Eyeliner	10	10(100.0)	72.5-100.0	NS	3(30.0)	10.8-60.3	

^a^Because of complete response, no significant diffeence was observed between cosmetic types.

**Table 2:** Microbial Counts (10^3^ CFU g^-1^) and association between contamination by bacteria and fungi in shared cosmetics available in women beauty salons

**Microorganisms**	**Powder**	**Cream**	**Mascara**	**Eyeliner**	***P*** ** value**
Bacteria					
*Acinetobacter*	300	350	320	NC	0.255
*Escherichia coli*	NC^a^	-	-	850	0.008
*Bacillus*	NC	230	320	500	0.802
*Pseudomonas*	208	195	180	125	0.576
*Staphylococcus*	960	544	410	144	0.518
*Streptococci*	NC	23	440	684	0.324
*Klebsiella*	-	-	21	-	0.233
*Citrobacter*	-	-	12	-	0.552
*Salmonella*	-	-	32	-	0.233
*Alcanigenes*	-	-	20	-	0.383
Fungi					
*Candida*	-	-	30	-	0.662
*Rhodotorula*	200	-	126	115	0.131
*Penicillium*	3.5	-	-	-	0.100

^a^Non-countable.

## Discussion

Results showed that all of the sampled cosmetics were contaminated by bacteria which is more than the rate of 63% reported from in-use individual cosmetics.^8^ Preservatives of the cosmetics remain active on the skin that might alter the skin microflora which are responsible for protection and supplying skin safety.^12^ Cosmetics are not produced in sterile condition and are often shared in beauty salons which could cause the increase of microbial contamination within cosmetics.^13^ Contamination level in our study is higher in comparison with a study in the United States that was conducted on 3000 shared cosmetic tester kits available to the public and reported 50% contamination of the products by bacteria.^14^

Contamination level in the powders was higher than the other cosmetics. It can be deduced that the powders are frequently in contact with air and also the common use of skin powder pads can cause the higher contamination rate. In addition, application of the natural ingredients in the formulation of powders including talc, Fuller’s earth and bentonite might increase the contamination level.^15^ Contaminated eye cosmetics, particularly mascaras, are associated with ocular infections.^5,16^ Our result revealed that in all the examined cosmetics, mascaras had a more bacterial diversity, because of its aqueous-based formulation and greater chance of bacterial deposits originating from the environment and from the surface of the eyelashes, which makes the product more susceptible to infections.^6,13^

About 19.2% (95% CI=10.8%-31.9%) of in-use cosmetics were contaminated by fungus and yeast. Fungus contamination ratio in cosmetics was low compared with bacteria. It can be attributed to the more ability of cosmetics preservatives in prevention of fungus growth.^17^ In this study, isolation of gram-negative bacteria was more than gram-positive bacteria, while gram-positive bacteria is more predominant in the skin flora.^17,18^ It can be concluded by the more resistance of gram-negative bacteria to severe condition which could cause growth of them in cosmetics. *Streptococci* species were the most dominant isolated bacteria, which also have been reported in personal cosmetics.^19^* Streptococcus* species can cause skin infections like Erythematous rash.^20^ *Pseudomonas* spp. was the most dominant species isolated from the eyeliners. *Pseudomonas aeruginosa* have been mostly reported in personal cosmetics.^2,5,21^ Because *Pseudomonas aeruginosa* is one of the natural skin microflora , it can be transferred to the cosmetics from consumer’s skin.^18^* Pseudomonas aeruginosa* can cause skin infections.^22^ Also, *Acinetobacter* was isolated from the examined cosmetic and as skin microflora play an important role in skin infections.^18,23^ *Bacillus* spp., *Staphylococcus* spp. and *Escherichia coli* were the other isolated bacteria from the cosmetic. *Staphylococcus* spp. causes skin infections such as acne and desquamate.^24^ *Bacillus* species are transient skin microflora. *Bacillus anthracis* causes focal necrotizing cellulitis in the skin. Use of eye cosmetics contaminated with* Bacillus cereus* causes severe eye infections.^24^ *Candida* and *Rhodotorula* also were isolated from the cosmetics. *Candida* has been reported in other personal toiletries studies.^5,25^ *Candida* plays an important role in the establishment of skin lesions, rash and dermatitis.^26^ Also in this study *Salmonella, Citrobacter, Klebsiella* and *Alcanigenes* have been isolated.

Higher density and diversity of bacteria isolated from shared cosmetics that obtained from beauty salons in comparison with personal cosmetics reported in the literatures.^3,5,21,27^ When several people share the same cosmetic an instance contamination may take place and because each individual has unique skin microflora that could be harmful to another person. The number of colony forming units of aerobic mesophilic microorganisms for eye and other cosmetics must not exceed 10^2^ and 10^3^CFU g^-1^ of products, respectively.^28^ In all the examined cosmetics available for public make-up in women beauty salons, the number of microbial counts was more than the maximum favorable level.

## Conclusion

Finally, our findings showed that microbial contamination rate in cosmetics which are shared in beauty salons is higher than the rates reported for personal cosmetics in the literatures. However, the hygienic condition of the salons, socioeconomic level of the area that the beauty salons are located and even individual health behaviors of the costumers could be impressive factors on the contamination rate of cosmetics which are not assessed in this study.

The skin microflora of anyone is distinctive and when several people share the same product, the rate of contamination could be increased. Therefore, it is suggested to avoid long-term use, share or use public cosmetics in toilet saloons and keep the used cosmetics in dry, cool, and fastened packets. Also, it is necessary to promote or make compulsory the use of individual cosmetic kits in the beauty salons, intensify the hygiene inspections from the beauty salons, monitor behavior of the barbers and implement continuous health education programs by the hygiene inspectors for the beauty salon workers.

## Acknowledgments

This study is part of an MSc thesis in Environmental Health Engineering, which was submitted to Tabriz University of Medical Sciences (5/53/4643-1393.08.14). We appreciate Research Vice-Chancellor of Tabriz University of Medical Sciences for their financial support. We thank all the beauty salon lords who participated in sampling of their cosmetic kits.

## Ethical approval

Procedures were approved by the Research Vice-Chancellor of Tabriz University of Medical Sciences review board.

## Competing interests

No conflict of financial and non-financial interest declared between the authors.

## Authors’ contributions

LD was involved in the conception of the study, performed data collection and the analyses and drafted the manuscript. RD was involved in the conception of the study, interpreted the results from the analyses, performed significant revisions, assisted in the revision of the manuscript and approved the final version of the manuscript.
